# HIV Disclosure to Infected Children Involving Peers: A New Take on HIV Disclosure in the Democratic Republic of Congo

**DOI:** 10.3390/children10071092

**Published:** 2023-06-21

**Authors:** Faustin Nd. Kitetele, Wenche Dageid, Gilbert M. Lelo, Cathy E. Akele, Patricia V. M. Lelo, Patricia L. Nyembo, Thorkild Tylleskär, Espérance Kashala-Abotnes

**Affiliations:** 1 Department of Infectious Diseases, Kalembelembe Pediatric Hospital, Kinshasa 012, Democratic Republic of the Congo; akelekat@yahoo.fr (C.E.A.); patlelo@yahoo.fr (P.V.M.L.); 2 Centre for International Health (CIH), Department of Global Public Health and Primary Care, University of Bergen, 5020 Bergen, Norway; thorkild.tylleskar@uib.no (T.T.); esperance.abotnes@uib.no (E.K.-A.); 3 Faculty of Psychology, University of Bergen, 5020 Bergen, Norway; wenche.dageid@uib.no; 4 Centre Neuro-Psycho-Pathologique de Kinshasa (CNPP), University of Kinshasa, Kinshasa 012, Democratic Republic of the Congo; mlelogilbert@gmail.com; 5 Programme National de Lutte Contre le SIDA, Kinshasa 012, Democratic Republic of the Congo; tricianyembo@yahoo.fr

**Keywords:** HIV, disclosure, children, adolescent, peer support, peer approach, sub-Saharan Africa

## Abstract

Appropriately informing HIV-infected children of their diagnosis is a real challenge in sub-Saharan Africa. Until now, there is no consensus on who ought to disclose and how to disclose. This paper describes the model for HIV status disclosure in which HIV-positive children/adolescents are informed about their diagnosis in a process conducted by young peers under healthcare worker (HCW) supervision in a hospital in Kinshasa, the Democratic Republic of Congo. This new take on HIV status disclosure involving peers includes four stages that help the trained peer supporters to provide appropriate counseling, taking into account the age and level of maturity of the child/adolescent: the preliminary stage, the partial disclosure stage, the full disclosure stage, and the post-disclosure follow-up stage. Of all children/adolescents whose HIV status disclosure data were documented at Kalembelembe Pediatric Hospital (KLLPH) between 2004 and 2016, we found that disclosure by peers was highly accepted by parents, children/adolescents, and health workers. Compared to children/adolescents disclosed to by HCWs or parents, children/adolescents disclosed to by peers had (a) fewer depressive symptoms reported, (b) better drug adherence resulting in higher viral load suppression, and (c) a higher proportion of survivors on treatment. We found that involving peers in the disclosure process of HIV is an important approach to ensure adherence to treatment, resilience, and mental wellbeing of HIV-infected children/adolescents.

## 1. Introduction

Globally, only 52% of children living with HIV are on life-saving treatment, far behind adults where 76% are receiving antiretroviral treatment (ART) [1]. Concerned by the stalling of progress for children, and the widening gap between children and adults, UNAIDS, UNICEF, WHO, and partners have brought together a global alliance to ensure that no child living with HIV is denied treatment by the end of the decade and to prevent new infant HIV infections [2].

It is, therefore, important to ensure that adolescents understand their diagnosis and their treatment regimen so that they can manage their health and successively take over the responsibility as they grow in age and maturity.

Unfortunately, disclosure of HIV status to children/adolescents is a controversial topic. Despite WHO recommendations of full disclosure by 12 years of age, the disclosure rates of HIV status disclosure to children/adolescents living with HIV in sub-Saharan Africa (SSA) are generally low, ranging from 9% to 72%, and constitute a significant public health challenge [3]. In SSA, parents are often reluctant to inform children of their HIV diagnosis because of fears of being blamed or the child inadvertently revealing the family’s HIV status to others, leading to stigmatization and isolation [4,5,6].

Delayed and nondisclosure of HIV status to ALHIVs led to a lack of awareness about the disease, nonadherence to ART, and unsafe sex practices that could increase the risk of HIV transmission and reinfection [7].

In DR Congo, as in other countries, where perinatal HIV infection is the main cause of HIV among children, the experience shows that parents are often psychologically affected by the fact that they are ‘responsible’ for the infection of their child. As such, some may give fragmented, incomplete, or false information to the child [8].

Despite the barriers, it is important to gradually bring necessary and sufficient information about HIV to infected children adapted to their level of understanding [9]. Eventually, full disclosure of HIV status is an essential part of a child’s healthcare. It is a sign of respect for their rights as an individual [10]. This is to avoid immediate and long-term consequences of the incidental discovery of HIV status among unprepared children/adolescents, which could lead to or exacerbate depression, worries, and other negative mental health outcomes.

Indeed, these negative outcomes impact the child’s quality of life, as well as their social and academic life, and they may also generate self-stigmatization [11]. The negative consequences also affect family life, including the parenting ability [12,13].

According to a study carried out in Kinshasa in the Democratic Republic of Congo (DR Congo) in 2010 among 259 HIV-infected children aged 5–17 years, only 3% of caregivers had informed their child of their HIV status [14].

In DR Congo, as in other countries in SSA, the rate of disclosure of HIV status to children and adolescents remains low, showing difficulty to reach the 95–95–95 targets of the UNAIDS by 2030 if new strategies are not adopted to address this low prevalence [15].

Studies on disclosure conducted in the DR Congo since 2007 have explored different approaches, as well as their advantages and disadvantages [14,16,17,18]. Using the disclosure process involving parents as modeled in high-income countries, we encountered several problems when parents were the ones leading the process of HIV status disclosure to children/adolescents in the DR Congo.

Thus, the medical staff at Kalembelembe Pediatric Hospital (KLLPH) thought that it was necessary to reconsider the disclosure approach and involve healthcare providers in the process. However, despite involving healthcare providers in the disclosure process discussions, the expected results on mental health and viral load were not optimal.

In 2014, in order to improve the disclosure process and increase the number of children/adolescents disclosed, a new take on HIV status disclosure was needed and, therefore, tried. This new disclosure process took into account the following: (1) cultural context of the DR Congo, because in the event of a difficult or challenging situation, the affected person is usually assisted by a person who has experienced the same situation, “a peer supporter” (e.g., on the death of a loved one, divorce, or failure in a given attempt); (2) the experience of peer support in mental health [19]; (3) generally, adolescents tend to resist any dominant source of authority, such as parents, and prefer to socialize more with their peers than with their families [10]; (4) research also suggests that adolescents are more likely to modify their behaviors and attitudes if they receive health messages from peers who face similar concerns and pressures [17]; (5) lack of trained staff to assign to the care of HIV-infected children.

Considering these challenges, the staff of KLLPH developed this project in order to improve the disclosure process among children in DR Congo.

Our objective was to explore whether the disclosure process taking into consideration the sociocultural dimension to the process of disclosure of HIV status by gradually integrating and implementing the “peer supporter approach” or “peer-to-peer approach” in the different stages would be beneficial.

## 2. Subjects and Methods

In this approach, the process of disclosing the HIV status to an infected child occurs in a progressive manner, taking into account the cognitive skills and emotional maturity of the patient. A team of individuals is utilized for the process:

1. Parents or caregivers,
2. Healthcare workers (HCWs), i.e., nurses, psychological care professionals, general practitioners, pediatricians, and psychiatrists,
3. Peers.

### 2.1. Definition of Peers for This Work

A peer educator is a member of a peer group that receives special training and information and tries to modify a person’s knowledge, attitudes, beliefs, or behaviors among the group members [20,21].

A peer supporter is someone with the lived experience of recovery from a health condition, substance use disorder, or both. They provide support to others experiencing similar challenges. They provide nonclinical, strength-based support and are “experientially credentialed” by their own recovery journey [22].

The relationship between the peer supporter and the recipient is characterized by trust, acceptance, understanding, and the use of empathy [23].

For this project, peer educators and supporters were volunteer adolescents or young persons living with HIV, aged 12–24 years old, who knew of and accepted their HIV status, had good adherence to treatment, and had been trained to support people living with HIV (PLHIV). The peer supporter tutored, counseled, supervised, guided, and supported the disclosure process under the supervision of HCWs.

### 2.2. Procedures

Stages of the process

Disclosure is a gradual process that involves many complementary steps ranging from simple information to complex information, generalities to specifics, and from partial to full disclosure. It is conducted in two phases, each split into two stages, for a total of four stages (Table 1).

Duration of the process

Disclosure is a complex and long-term process. The exact duration varies from one child or adolescent to another. It also depends on their maturity and cognitive development. The peer and HCWs should establish an individualized or collective HIV status disclosure plan in agreement with the parents/tutors. This happens during parent preparation sessions for the disclosure of their children.

Assessment of cognitive skills and emotional maturity of the patients

The Diagnostic Interview for Children and Adolescents (DICA) was used as a tool to gather information on the emotional, cognitive, or behavioral situation of the children [24]. The cognitive information collected made it possible to classify the patient according to the stage of cognitive development described by Jean Piaget. Each stage of cognitive development was then correlated with a stage in the disclosure process [25].

**Table 1:** Stages of the process of HIV disclosure to children/adolescent.

	PHASE I or Pre-Disclosure		PHASE II or Full Disclosure and Follow-Up	
	Stage 1 or Preliminary Stage	Stage 2 or Partial Disclosure	Stage 3 or Full Disclosure	Stage 4 or Post-Disclosure Follow-Up
**Aim**	To develop a climate of trust, openness, frank dialogue, and empathy and involve the child/adolescent in their care	To help the child/adolescent to discover and understand the chronic nature of their condition and the importance of treatment	To make the child/adolescent discover and accept that their chronic illness is HIV (named HIV)	To assess possible psychological reactions, and to offer child/adolescents support multifaceted (social, emotional, etc.)
**Age**	Less than 6 years old or more ^a^	6–12 years or more ^a^	8–12 years or more ^a^	8–12 years or more
**Schedule**	Once a month for about 3 h	Once a month for about 2 h or as needed from case to case	When the child is ready; the duration depends on the child	Once a month, for at most 2 h or as needed from case to case
**Methodology**	Two parts: recreational and training Provided remotely through digital support (videoconference or WhatsApp) when access to the hospital was not possible	Metaphoric tools (on the functioning of the human body, causes of diseases, acute and chronic diseases, etc.), dialogue, discussion, reading, homework, and internet research Provided remotely through digital support	Peer/HCW ensures that the child/adolescent has understood the following: - their illness is chronic in nature - adherence and privacy are important Peer adopts a procedure that allows the adolescent to discover for themself their chronic illness or guide the interview so that together they come to a conclusion about HIV	Mental health post-disclosure follow-up assessment by the following tools: - PHQ-2/PHQ-9 ^b^ [26,27] - HAM-D ^c^ [28] - HAM-A ^d^ [28] - PTSD ^e^ [29] Provided remotely through digital support Inclusion in the self-support group
**Role of peers**	The peer prepares the child/adolescent for the ultimate disclosure and instills in them the notion of confidentiality	Peers help adolescents to find by themselves or, with the help of other people, the names of the different diseases and to classify them according to whether they are acute or chronic, infectious, or noninfectious The peer emphasizes the difference between HIV (infection) and AIDS (disease), the prevention and management of opportunistic infections, and the role of ARTs	Before the full disclosure, the Peer/HCW ensures the following: - the level of cognitive development of the child/adolescent allows it to be disclosed - their parents agree to the full disclosure - who should provide full disclosure and what is the ideal time to disclose status Proceed to full disclosure (confirm and name the HIV)	The peer helps the child/adolescent to join the self-help group (SSG); the SSG helps them to do the following: - discover that they are not the only one to have HIV - receive social support to live with HIV - learn positive life skills to face HIV - learn strategies to deal with thoughts and emotions that might arise The SSG is an additional way to detect problems that the person disclosed to might have
**Modality**	Group or individual session	Group or individual session	Group or individual session	Group session
**Session lead**	Peer under the supervision of HCW	Peer under the control of HCW	Peer under the control of HCW	Peers under the supervision of HCWs
**Particularity**	The peer makes the child/adolescent aware of the importance of hygiene, nutrition, prevention against infections, and the impact of chronic illnesses on the individual and society	At this stage, the peer informs the adolescent that they have a chronic illness, and that the disease can be controlled by treatment The peers share their experience of also carrying a chronic disease without revealing the name of the disease The peers talk about the bad experiences of nonadherence to treatment and its effects The peer deconstructs negative images and stigma related to chronic diseases such as HIV, diabetes, asthma, and others	At this stage, the peer offer three types of support based on experiential knowledge: emotional, appraisal, and informational [30] The peer (group of peers) reveals their identity and shares their own experiences as a PLHIV with the child/adolescent disclosed to. With the existing empathy, they give appropriate advice they provide comfort and insist on adherence to treatment and confidentiality They fix appointments and keep in contact	The self-support group meeting of peers is a place for exchange, sharing experiences, and mutual listening, which encourages free expression without fear of judgment from others or stigmatization; it promotes solidarity between participants and develops and mobilizes the resources necessary to face reality Peer/HWCs use interviews, home visits, telephone calls or WhatsApp, and email Reference to a mental health specialist in the event of clinically significant symptoms

^a^ Depending on the maturity level. ^b^ Patient Health Question (PHQ-2/PHQ-9 modified for Adolescents (PHQ-A)) screening for major depressive symptoms. ^c^ The Hamilton Depression Rating Scale (HAM-D): screening for major depressive symptoms. ^d^ Hamilton Anxiety Rating Scale (HAM-A): screening for anxiety symptoms. ^e^ Internalizing behavior problems and post-traumatic stress disorder (PTSD).

## 3. Results

Between 2004 and 2016, 802 HIV-infected adolescents were enrolled in care at the KLLPH. Among them, less than half benefited from the disclosure of their HIV status, of which 244 were documented.

The results of the process of HIV status disclosure to children, as carried out at the KLLPH, have been described in several publications [14,16,17,18,31,32]. The noted advantages and disadvantages realized during the process are presented in Table 2.

Of the 244 adolescents living with HIV (ALHIV), aged 10–19, whose HIV status disclosure data were documented at KLLPH between 2004 and 2016, 142 (58.2%) were females, the mean age at the disclosure was 15.3 years (SD: 2.3), all were infected by mother-to-child transmission, and only 17.6% of them had both parents alive. Of these, 131 were disclosed to by HCWs, along with 71 by parents/guardians and 42 by peer supporters.

The overall prevalence of depressive disorder post-HIV status disclosure was 36% using Patient Health Question (PHQ-2/PHQ-9 modified for Adolescents (PHQ-A)) screening for major depressive symptoms and the Hamilton Depression Rating Scale (HAM-D): screening for major depressive symptoms (Table 3). In presenting the results stratified in terms of the type of disclosure, we found that the difference was significant between adolescents disclosed to by peers and those disclosed to by healthcare workers or parents with regard to depression (10% vs. 26% and 69%), suppressed viral load (67% vs. 41% and 36%), and death (2% vs. 17% and 6%)

## 4. Discussion

The present article describes a new approach to disclosing the HIV status of children and adolescents involving peers, which significantly improved the outcomes for the adolescents in terms of quality of life, treatment adherence, and health.

Our data report an overall depression prevalence of 36% in post-HIV-status disclosure adolescents. This result aligns with the median point prevalence for depression reported in a recent systematic review, i.e., 22.2% (IQR 15.5–41.1) in sub-Saharan adolescents living with AIDS [33] and 41.7% in a recent study conducted in Spain [34].

Moreover, our findings show that adolescents disclosed to by peers had a significantly lower prevalence of depression than those disclosed to by healthcare workers and parents (10% vs. 26% and 69%). The difference was also significant with regard to suppressed viral load, loss to follow-up, and death.

These findings are consistent with previous systematic reviews showing that the prevalence of mental health problems may vary according to the type of informant [35].

As also described in a systematic review, but in another context [36], a peer approach or peer-self-support group had a positive psychological effect on reducing depression, improving quality of life and care of ALHIV disclosed to. ALHIVs who are disclosed to of their status are four times more likely than nondisclosed adolescents to adhere to ART [37].

The result of the study revealed that ALHIV disclosed to by parents or healthcare workers had poor adherence to treatment, behavioral problems, loss to follow-up, and early death. These issues arose, at least in part, because some disclosures were performed inappropriately, incompletely, or accidentally. Some studies conducted in the region also found that HIV-positive parents were challenged with disclosure to their children [18], and others found that healthcare workers and parents had minimally tailored guidance on how to approach the issue of disclosure to adolescents [37].

As described in a recent systematic review, ALHIV who are informed of their HIV status either through parents or accidentally through healthcare workers or other family members demonstrated a negative effect, with some feeling anxious and others becoming depressed [38]

As recently published in bibliometric analyses, depression is not only a clinical disorder but also affects adherence to ART; it weakens the effectiveness of treatment and even increases the mortality rate of patients [39].

In line with studies supporting the involvement of peers, we also found that the continuum of care through peer support helps children/adolescents disclosed to feel supported, guided, and understood, and it reduces stigmatization and improves adherence to treatment. Children/adolescents disclosed to will continue to benefit from follow-ups in the form of home visits, support groups, social networks, recreational activities, telephone contacts, and counseling. These findings justify the traditional Congolese pedagogy which teaches “peer support” through the “image of the banana/plantain trees”. A banana tree is filled with water and fibers, and it is so fragile that it cannot stand alone to carry bananas. Thus, banana trees always grow in groups to support each other in carrying the weight of banana fruits. In this way, the banana trees can withstand strong winds without collapsing. Through the symbolism of banana trees, peers help their fellow peers to accept carrying their burden with advice, to support each other in order to resist temptations and face adversity.

We found that our approach involving peers in the disclosure process of HIV facilitated resilience and acceptance, and improved the quality of life [31,32].

The person disclosed to and the peer share similar conditions and challenges, leading to an the inhibition of the “inhibiting effect”, favoring (1) open and frank dialogue, (2) understanding, (3) and trust [40]. Thus, the social sharing of emotion greatly allows the emergence of demeanors that are characteristic of the relationship of attachment [41]. It is an empathy dimension whereby the peer expresses empathy, understanding, and support. This empathic dimension can easily lead to trust and help the disclosed child/adolescent to open up with confidence. The socio-cognitive dimension increases understanding and ability to deal with the disease.

In their role as a mentor, the peer is trained to provide children/adolescents with appropriate information on HIV/AIDS, which will play a determining role in the positive perception of HIV disease and improving decision making and sustainable behavior of PLHIV. Our findings are in line with previous studies that reported a socio-cognitive dimension in the process of involving peers because, through empathy, peers reinforce confidence and trust [36]. The socio-cognitive dimension confirms the assertion that social sharing oriented toward cognitive processing promotes emotional recovery that would help to improve psychological resilience [42,43].

Involving peers in the disclosure process of HIV constitutes an important approach in the context of HIV in DR Congo to ensure adherence to treatment, resilience, and mental wellbeing of infected children/adolescents.

### Strengths and Limitations

The present study had several strengths because it is the first study in the DR Congo and sub-Saharan Africa to investigate a different approach to improve HIV disclosure among children and adolescents involving peers. This approach was based on taking into account different factors that were believed to have condemned the disclosure process by parents and healthcare workers to failure according to feedback from the children/adolescents disclosed to and their parents.

As part of task shifting and differentiated care, peer support may ease the tasks of HCWs, increase the rate of disclosure, and improve compliance to treatment. It is a lower-cost task-shifting model.

Our study is the first to demonstrate that the peer-to-peer approach in our context improves the disclosure process of the HIV status of HIV-infected children/adolescents, their mental wellbeing by minimizing mental health symptoms, and their access to care. It has the benefit of involving peers and being widely accepted, as reported by children/adolescents and their parents. The DRC is facing a lack of mental health workers, and involving peers can contribute to accessibility to care at a low cost in the context of a lack of health professionals.

Another strength of this study is that it is an overview of the benefits of involving peers in the disclosure process taking into consideration different important aspects such as the medical history, stages of the disease, and perception of the HIV disclosure process as perceived by children/adolescents and their parents.

This study also presented challenges and limitations. Our study was limited to a single site, Kalembelembe Pediatric Hospital, and its generalizability cannot be assessed.

Another limitation to be considered is the law requiring prior parental consent before disclosing HIV status to an ALHIV unless there is a compelling reason. Unfortunately, up to half of the parents did not consent to any kind of HIV status disclosure because of fear of the child’s reaction, with the risk of adolescents becoming sexually active without knowing their HIV status. It was also noted that the peer approach does not take into consideration adaptations or adjustments of the process to suit children/adolescents with cognitive disabilities.

Although our study had some limitations, it generated data that can serve as a basis to better understand and improve the disclosure process in a low-income country with limited resources. Further studies at a larger scale are needed to look deeper into other advantages and limitations of this approach in order to support children/adolescents and their families to improve the disclosure process of affected children and to extend the peer approach to other chronic diseases of children/adolescents.

## 5. Conclusions and Health Implications

Involving peers in the disclosure process of HIV is an important approach to ensure adherence to treatment, resilience, and mental wellbeing of HIV-infected children/adolescents.

This approach has implications for the management of HIV among youth in the DR Congo. Firstly, it improved compliance with treatment, follow-up, and life expectancy. Secondly, this approach has been implemented at the national level by the Ministry of Health as the new approach for HIV disclosure among children in the DR Congo.

## Figures and Tables

**Table 2:** Advantages and disadvantages for the different parties of involving peers in the HIV disclosure process for children/adolescents [14,16,17,18,31,32].

	Advantages	Disadvantages/Dangers
**For the** **child/adolescent disclosed to**	- The peer presents themself as a model person and, therefore, an example to follow - The peer is often considered a mentor and becomes the interface in front of concerns or questions - The proximity of the age brings down barriers and promotes a frank dialogue - Attachment to the peer allowing real-time monitoring - Fewer depressive symptoms - Adherence and retention of care - Could become autonomous and develop self-esteem	- Bad disclosure (the child is not fully prepared for announcement) - Inappropriate or involuntary disclosure (child disclosed under the influence of anger or inadvertently) - Accidental disclosure (child informed of their illness purely accidentally) - Mental health issues (anxiety, PTSD, addiction, etc.)
**For the peer**	- Feels valued by the confidence and the role played - Improves their adherence to treatment because they must remain a model for others - By giving advice, they also change behavior - Plays the role of mentor for certain adolescents - The skills learned as a trained peer supporter help them to maintain their personal resilience - Volunteers feel clearly valued and useful in society; they ensure permanence in turn at the KLLPH according to their timetable (school program)	- Behaving like a healthcare provider - Lack of time for themself (extra work) - Having significant responsibilities - Taking advantage of empathy to abuse others (false empathy) - Neglecting education and studies for the benefit of the support of peers - Becoming too emotionally involved
**For the parents**	- Less fear - Less stress - Frank and open dialogue with the child - Parents/guardians whose children/adolescents are peers support them and accompany them in this task by their advice and means of transport	- Do not support the process - Fear of the community’s reaction (stigma and discrimination) - Shame, guilt, and fear of blame when disclosing the status of their perinatally infected children
**For the healthcare worker**	- Successful task-shifting model - Work alleviation - Priorities for patients most in need of care following differentiated care	- Fear or failing - The disclosure process is taken as extra work, i.e., supervising peers and assessing disclosed children
**For the institution**	- Less loss to follow-up and improved adherence - Fewer admissions due to complications of HIV/AIDS - Alleviation of healthcare worker tasks - Fewer burdens: the participation of unpaid volunteers makes peer education less expensive - Promote good collaboration between the institution and peers	- Lack of control of the situation if peers are not compliant - Unavailability of meeting rooms or spaces dedicated to young people

**Table 3:** Depressive symptoms, viral load suppression, and treatment outcomes in relation to who conducted the full HIV disclosure to the child/adolescent.

	Total of Adolescents N = 244	Disclosed to by Health Worker *n* = 131	Disclosed to by Parents *n* = 71	Disclosed to by Peers *n* = 42	*p*-Value
	N (%)	*n* (%)	*n* (%)	*n* (%)	
Depressive symptoms					0.000 *
Not depressive	157 (64)	97 (74)	22 (31)	38 (90)	
Depressive	87 (36)	34 (26)	49 (69)	4 (10)	
Viral load (VL)					0.007 *
VL suppressed	99 (41)	47 (36)	24 (34)	28 (67)	
No VL suppressed	102 (42)	62 (47)	29 (41)	11 (26)	
Missing	43 (18)	22 (17)	18 (25)	3 (7)	
Outcome					0.011 *
Alive	189 (78)	101 (78)	50 (70)	38 (93)	
Deceased	21 (9)	8 (6)	12 (17)	1 (2)	
Lost to follow-up	32 (13)	21 (16)	9 (13)	2 (5)	

* Statistically significant difference between groups.

## Data Availability

Data supporting reported results can be requested from the corresponding author upon reasonable request.
